# Anemia diagnosis and therapy in malignant diseases: implementation of guidelines—a representative study

**DOI:** 10.1007/s00520-023-08267-4

**Published:** 2024-01-19

**Authors:** Hartmut Link, Markus Kerkmann, Laura Holtmann, Markus Detzner

**Affiliations:** 1 Internal Medicine, Hematology and Medical Oncology, D-67661 Kaiserslautern, Germany; 2 MMF GmbH, Lindberghweg 132, D-48155 Münster, Germany; 3 AIO-Studien-gGmbH, Kuno-Fischer-Straße 8, D-14057 Berlin, Germany; 4 Working Groups Supportive Care (AGSMO), Medical Oncology (AIO) of the German Cancer Society, Berlin, Germany

**Keywords:** Anemia diagnostics, Transfusion, Iron therapy, Erythropoiesis-stimulating agents, Patient blood management

## Abstract

**Purpose:**

Anemia in cancer should be diagnosed and treated according to guideline recommendations. The implementation of ESMO and German guidelines and their effect on anemia correction was analyzed.

**Methods:**

This retrospective epidemiological study, representative for Germany, analyzed data on anemia management of cancer patients with anemia ≥ grade 2. The Guideline Adherence Score (GLAD) for diagnosis (GLAD-D) and therapy (GLAD-T) was defined as follows: 2 points for complete, 1 point for partial, 0 point for no adherence.

**Results:**

Data were analyzed for 1046 patients. Hb levels at diagnosis of anemia were 8–10 g/dL in 899 (85.9%) patients, 7–8 g/dL in 92 (8.7%), and < 7 g/dL (5.0%) in 52. Transferrin saturation was determined in 19% of patients. Four hundred fifty-six patients received RBC (43.6%), 198 (18.9%) iron replacement, 106 (10.1%) ESA, and 60 (5.7%) vitamin B12 replacement. 60.6% of patients receiving iron replacement were treated intravenously and 39.4% were treated orally. Two hundred eighty-eight (36.6%) of 785 patients receiving transfusions had no guideline-directed indication. GLAD-D was 2 in 310 patients (29.6%), 1 in 168 (16.1%), and 0 in 568 (54.3%). GLAD-T was 2 in 270 patients (25.8%), 1 in 320 patients (30.6%), and 0 in 456 patients (43.6%). Higher GLAD-D significantly correlated with higher GLAD-T (τB = 0.176, *p* < 0.001). GLAD-T 2 was significantly associated with greater Hb increase than GLAD-T 0/1 (*p* < 0.001) at 28 days (10.2 vs. 9.7 g/dL) and at 2 months (10.4 vs. 9.9 g/dL).

**Conclusions:**

Anemia assessment is inadequate, transfusion rates too high, and iron and ESA therapy too infrequent.

**Trial registration:**

ClinicalTrials.gov, NCT05190263, date: 2022–01-13.

## Introduction

Anemia is the most common comorbidity of malignant diseases, which may increase with diagnostic and therapeutic interventions. Patient’s symptoms, complaints, and quality of life correlate with the degree of anemia [33]. Depending on tumor type and stage, the prevalence of anemia is 31–50% in untreated solid tumors; the prevalence is even higher in hematologic neoplasms [37]. Anemia is also unfavorable for the course and therapy of malignant disease and of survival [14, 28, 29, 33, 39, 48].

In terms of supportive care, all oncologists need to address the diagnosis and treatment of anemia in cancer [30]. ESMO and national societies provide evidence-based guidelines for the situational diagnosis and management of anemia. They provide specific decision support algorithms for analyzing the causes and treatment of anemia [1].

Therefore, it is of great interest to analyze if and how these guidelines are implemented and to assess the relevance of guideline adherence. The aim of this study was to evaluate the implementation and adherence to the ESMO Anemia Guidelines [1], the DGHO Onkopedia Germany [26], the German guideline on supportive care [31, 44] and the cross-sectional guidelines of the German Medical Association [16] in clinical routine in patients with solid tumors and malignant lymphomas at high risk of anemia. To this end, a patient documentation was conducted to observe the current quality of anemia diagnostics, therapy, and guideline implementation in hospitals and among oncology practices. In addition, the effect of guideline implementation on anemia correction was to be investigated.

## Methods

A retrospective analysis was performed on a representative sample of patients from outpatient and inpatient settings in Germany. The documentation took place in Q3 and Q4 /2021.

In total, 1395 institutions were contacted. All certified centers were contacted, as well as all institutions that had participated in at least phase 1 (health care structure analysis) of the previous AIO AG Supportive Therapy and AGSMO studies. Three hundred eleven centers participated in phase 1 and provided feedback on their patient volume in the mentioned indications (response rate 22.3%).

One hundred forty-three centers participated in patient documentation (phase 2). The patients to be documented were selected in a representative way on the basis of the previously performed health care structure analysis data and distribution ratios from phase 1. The participating centers were assigned to four clusters (each subdivided by disease and hospital/practice) according to the number of patients to be documented: centers with very high, high, medium, and low patient volume (PV). Cutoffs for the distribution were determined using the 75%, 50%, and 25% quartiles of PV. To avoid selection bias and ensure random selection, centers were asked to document all patients meeting the inclusion criteria from the cutoff date (01/01/2021) until the predefined number of patients in this center was reached. Thus, the real care situation is proportionally and representatively reflected in the sample.

### Patients

From the cutoff date (01/01/2021), all patients meeting the following inclusion criteria were chronologically documented per center until the predefined set of patients for that center was reached:

- Adults ≥ 18 years of age
- Diagnosis of anemia grade ≥ 2 (CTCAE 5.0), resp. an Hb value < 10 g/dL (or < 6.2 mmol/L) between 01/01/2021 and 06/30/2021
- Diagnosis of gastrointestinal cancer (colon, rectum, stomach, esophagus, or pancreas), breast cancer, lung cancer (NSCLC or SCLC), or malignant lymphoma (non-Hodgkin’s lymphoma or Hodgkin’s lymphoma)

Patients were excluded if:

- The follow-up period was less than 4 weeks and if there was no contact with the patient after the diagnosis of anemia
- Diagnosis of a bone marrow disease (e.g., MDS, CML, CLL)

Patient characteristics, disease parameters, therapies, and lines of therapy were documented.

### Diagnostics

The following parameters were considered necessary for an adequate diagnosis of anemia and iron metabolism: hemoglobin, mean corpuscular volume (MCV), percent hypochromic red cells (%HRC), reticulocyte hemoglobin content (CHr), serum ferritin (SF), transferrin (TF), transferrin saturation (TSAT), and soluble transferrin receptor (sTfR). In addition, folic acid and B12 should be determined if needed.

The following definitions were used [1, 3, 50]:

- Iron deficiency (ID): TSAT < 20% or SF < 100 ng/mL
- Absolute ID (AID): SF < 100 ng/mL
- Functional ID (FID): TSAT < 20%, SF ≥ 100 ng/mL
- No ID: TSAT ≥ 20%; SF ≥ 100 ng/mL
- Signs of anemic hypoxia: tachycardia, hypotension, ECG ischemia, lactic acidosis
- Risk factors: coronary artery disease, chronic heart failure, cerebrovascular insufficiency

### Treatment

Guideline-compliant anemia management was defined by the following criteria [16, 44]:

- Restrictive red blood cell (RBC) transfusion policy:
  - Hb ≥ 10 g/dL: no RBC transfusion indicated
  - Hb ≥ 8– < 10 g/dL: RBC transfusion indicated only if there is evidence of anemic hypoxia
  - Hb ≥ 7– < 8 g/dL: RBC transfusion indicated:
    - Yes: in the presence of signs of anemic hypoxia
    - Yes: in the presence of other risk factors/insufficient compensation
    - No: no risk factors and adequate compensation
  - Hb < 7 g/dL: RBC transfusion indicated
- *Iron replacement and ESAs in patients receiving chemotherapy*:
  • Oral iron replacement is not recommended and should only be considered in patients with ferritin < 30 ng/mL, non-inflammatory disease (C-reactive protein (CRP) < 5 mg/L) and only in patients in complete remission.
  • Intravenous (IV) iron replacement in case of ID.
        - Absolute ID: IV iron and ESA if Hb remains < 10 g/dL during follow-up.
        - Functional ID: IV iron and ESA.
      • No existing ID.
           - ESA and IV iron if ID during follow-up
- *Iron replacement in patients not receiving chemotherapy:*
  • Oral iron replacement is not recommended and should only be considered in patients with ferritin < 30 ng/mL, non-inflammatory conditions (CRP < 5 mg/L) and only in patients in complete remission.
  • IV iron replacement in case of ID.
  • Absolute or functional ID: IV iron.
  - ESAs are only recommended in patients receiving chemotherapy.
  - Appropriate substitution in case of proven vitamin B12 or folic acid deficiency.

### Guideline adherence score (GLAD)

Based on our previous definition, a three-point GLAD score scale has been defined for diagnosis (GLAD-D) and treatment (GLAD-T), respectively [34].

1. **GLAD-D**
  - 2 points: diagnosis of anemia according to guidelines. Diagnosis of vitamin B12 or folate deficiency; if no vitamin B12 or folate deficiency: differential diagnosis of iron metabolism (SF + TF or TSAT or %HRC or CHr)
  - 1 point: no differential diagnosis. Low MCV without further differential diagnosis; normal MCV + normal SF without further differential diagnosis; normal vitamin B12 and folate levels, no further differential diagnosis
  - 0 points: lack of anemia diagnosis. Normal or elevated MCV and no vitamin B12 or folate diagnostics and/or no differential diagnosis of iron metabolism
2. **GLAD-T**
  - 2 points: therapy of anemia according to guidelines. AID/ID: IV iron or oral iron if CRP < 5 mg/L (+ / − ESA in chemotherapy); FID: ESA (only in chemotherapy) + IV iron or oral iron if CRP < 5 mg/L; no ID: ESA (only in chemotherapy) if no vitamin B12 or folate deficiency; vitamin B12 or folate deficiency: vitamin B12 or folate substitution
  - 1 point: failure to fully follow the guidelines. RBC with indication as primary and only anemia therapy; FID: ESA only (in chemotherapy) or iron replacement only; Hb ≥ 8 g/dL: no RBC, no other anemia therapy
  - 0 points: no adherence to guidelines. RBC without indication as primary and only anemia therapy (excluding RBC directly related to major surgery); Hb < 8 g/dL: no anemia therapy; ID: oral iron replacement and CRP > 5 mg/L; vitamin B12 or folate deficiency without replacement. ESA without chemotherapy

### Statistical methods

Statistical data analysis was performed using R version 4.2.2 [43]. Descriptive statistics include absolute and relative frequencies for qualitative characteristics; for continuous characteristics, location measures were calculated with corresponding measures of dispersion (median with interquartile range and minimum and maximum).

An analysis of variance for repeated measures (rmANOVA) was performed to analyze the increase in Hb after the diagnosis of anemia or the start of anemia therapy. Since the prerequisite for this measurement is a normal distribution of the measurement results, this was analyzed by histograms and Q-Q plots confirming the normal distribution. The second requirement for multiple measurements is sphericity. This was checked with the Mauchly test. Since sphericity is not present in the sample, the Greenhouse–Geisser correction was used to correct for sphericity violations.

To compare interval-scaled variables, such as GLAD scores, the Mann–Whitney *U* test [38] was performed when the independent variables were binomial. Kendall’s tau-b test (τB) was performed to examine the correlation between two continuous or interval-scaled variables [32].

Two multivariable logistic regression models were constructed to analyze which patient characteristics and center parameters correlated with guideline-directed anemia therapy. Because Hb increase proved to be relatively similar between GLAD-T 0 and GLAD-T 1, but significantly different from total guideline adherence, the analysis here was binomial (GLAD-T 0/1 vs. GLAD-T 2). For effect estimates from multivariable logistic regression, the corresponding odds ratios (OR) with associated 95% confidence intervals (95% CI) are reported. The significance level is two-sided with *p* < 0.05.

## Results

A total of 1046 out of 1053 documented patients could be evaluated. Five patients did not meet the inclusion criteria; two patients were double documented and were excluded.

Relevant patient and iron metabolism diagnostic data are summarized in Table 1.

**Table 1:** Patient characteristics at diagnosis of anemia and iron diagnostics performed

	Breast cancer	Gastrointestinal cancers	Lung cancer	Malignant lymphoma	Total
*Number of patients*	311	371	260	104	1046
*Age—years, median (25% and 75% percentiles)*	60 (51–69)	69 (61–76)	67 (61–74)	70 (57–79)	79
*Gender (male*–*female) %*	0.3–99.7	60.9–39.1	59.2–40.8	47.1–52.9	41.1–48.9
*Disease stage %: UICC, Ann Arbor in lymphoma*					
*N/A*^*a*^	1.0	5.4	–	–	
*I*	19.6	18.6	1.2	9.6	
*II*	38.3	25.6	8.5	20.2	
*III*	16.7	50.4	26.9	22.1	
*IV*	24.4	5.4	63.5	48.1	
*ECOG status (%)*					
*0*	38.3	19.1	18.8	26.0	25.4
*1*	36.3	46.4	50.0	41.3	43.8
*2*	11.6	15.4	18.8	7.7	14.3
*3*	2.6	3.2	1.9	5.8	3.0
*4*	0.0	0.0	0.0	0.0	0.0
*Unknown*	11.3	15.9	10.4	19.2	13.5
*Tumor therapy (%)*					
*Major surgery*	53.4	53.4	32.3	24.0	45.3
*Drug-based therapy*	98.1	87.6	92.3	92.3	92.4
*Radiation*	43.7	18.1	42.3	3.8	30.3
*Hemoglobin g/L, median (25% and 75% percentiles)*	9.4 (8.7–9.8)	9.2 (8.6–9.6)	9.3 (8.5–9.7)	9.2 (8.4–9.6)	9.3 (8.6–9.7)
*Iron deficiency diagnostics in % of patients*					
*MCV*	94.2	97.6	98.5	93.3	96.4
*Serum ferritin (SF)*	24.8	29.4	23.5	28.8	26.5
*Transferrin saturation (TSAT)*	19.9	22.1	16.9	15.4	19.5
*Hypochromic erythrocytes %HRC*	3.2	3.5	1.9	4.8	3.2
*Reticulocyte hemoglobin (CHr)*	3.9	6.5	7.3	9.6	6.2
*Soluble transferrin receptor*	0.3	2.4	0.8	1.0	1.2

^a^Three patients had no initial staging and were staged ypT0 N0 M0 after neoadjuvant chemotherapy

In addition, at the time of anemia diagnosis, vitamin B12 was determined in 8.1% of the patients and folic acid in 7.4%; at the follow-up of up to 2 months, these diagnostics were still performed in 5.6% of the patients.

Since serum ferritin is not well suited to assess iron status in tumor patients, it is better to determine transferrin saturation. This was done in only 19.5% of the patients. MCV is typically decreased in ID. Since the prevalence of individuals with decreased MCV in thalassemia is low in Germany, this parameter is diagnostic for ID.

Since MCV is always available at the time of blood count, we investigated whether there is a relationship between MCV and TSAT.

At the time of diagnosis (*n* = 204), the correlation coefficient for TSAT and MCV indicates a moderate relationship (τB = 0.256, *p* < 0.001). If only patients with iron supplementation and without RBC transfusion are considered, the correlation is even stronger (τB = 0.363, *p* < 0.001), but the number of patients is smaller (*n* = 72). There is no measurable effect in patients who did not receive iron supplementation or RBC transfusion (τB = 0.103, *p* = 0.182).

There is an association between reticulocyte Hb and TSAT, at the time of diagnosis (τB = 0.606; *p* < 0.001) and over all time points (τB = 0.476; *p* < 0.001). However, the samples are very small (time of diagnosis *n* = 21 and all time points *n* = 41) because reticulocyte Hb was rarely measured.

Red blood cell (RBC) transfusions were given to 456 (43.6%) of the patients, of whom 247 (23.6%) received RBCs once, 97 (9.3%) twice, and 112 (10.7%) received RBCs three or more times. One unit of RBCs was administered in 28.3% of transfusions, and two or more units of RBCs were administered in 71.7% of transfusions.

**Fig. 1:**
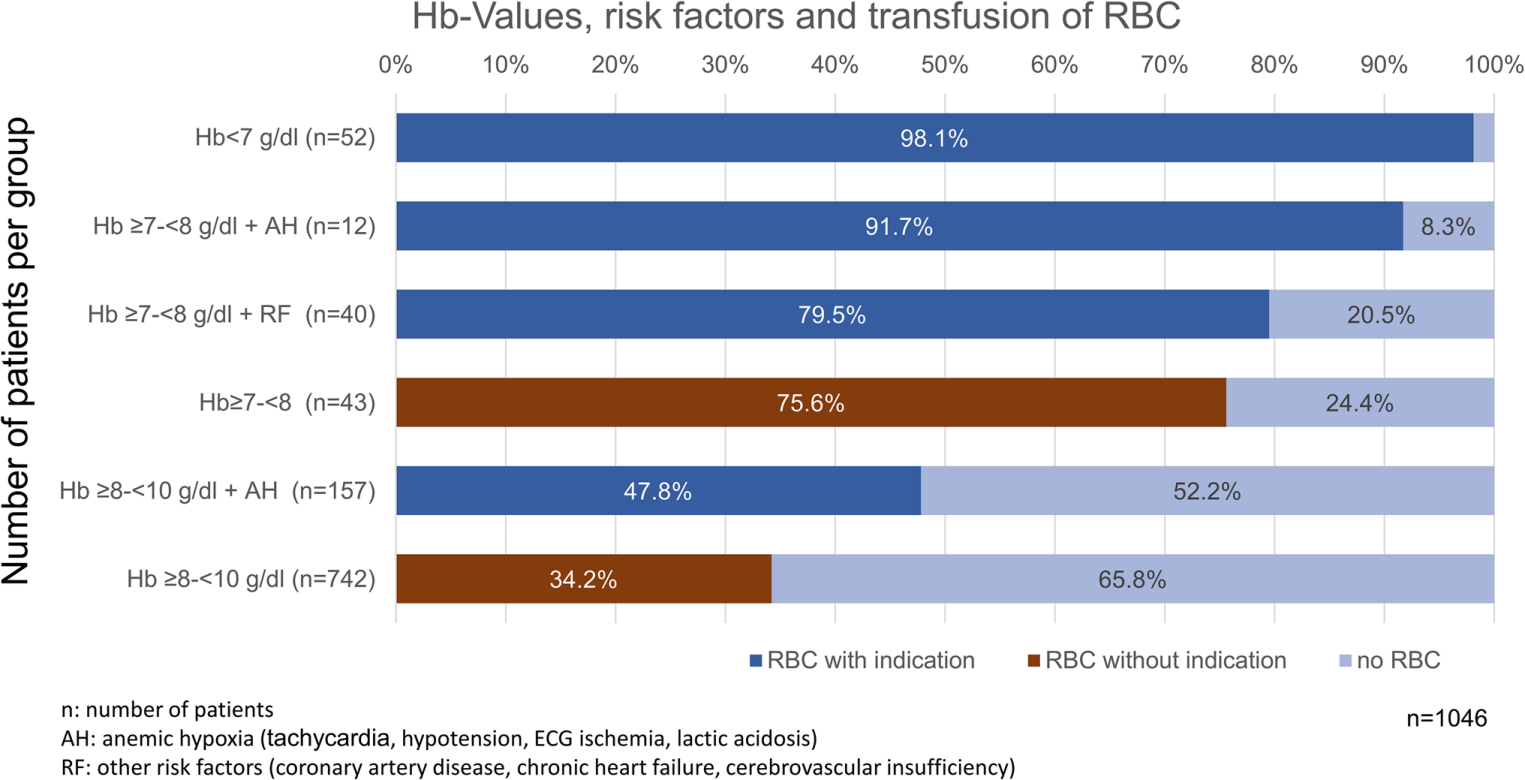
Transfusion of RBC and indication according to guidelines

The relationship between the number of RBC transfusions performed, taking into account Hb level and risk, and the number of transfusions indicated by guidelines is shown in Fig. 1.

In further analysis, 288 (36.6%) of 785 patients received a red blood cell transfusion without a guideline-directed transfusion indication. In terms of RBCs administered, this means that 63.2% of patients who received RBCs (*n* = 456) had no indication. Patients without a guideline-directed indication were also more likely to receive two or more units of RBCs (75.4%) than those with an indication (65.8%). Among all transfused patients (*n* = 456), 54.2% received one transfusion and 45.8% received two or more transfusions.

45.2% of the patients with an indication for transfusion (*n* = 166) had one transfusion occasion and 54.8% had two or more occasions. Among patients without an indication for transfusion (*n* = 288), 59.4% received one transfusion and 40.6% received two or more transfusions.

ID was diagnosed in 304 (29.1%) patients, no ID was present in 320 (30.6%) patients, and the diagnosis was incomplete in 422 (40.3%) patients. One hundred twenty patients received intravenous iron therapy and 78 patients received oral iron therapy. The inflammatory parameter (CRP) was above 5 mg/dL in 51 (42.5%) patients on IV iron and 19 (24.4%) patients on oral iron. The use of medical therapy with iron and ESAs is shown in Table 2 and was most common in patients with breast cancer and least common in patients with malignant lymphoma.

**Table 2:** Medical therapy in anemia

	Breast cancer *N* = 311		Gastrointestinal cancers *N* = 371		Lung cancer *N* = 260		Malignant lymphomas *N* = 104		Total *N* = 1046	
	*N*	%	*N*	%	*N*	%	*N*	%	*N*	**%**
*Iron substitution*	97	31.2	69	18.6	27	10.4	5	4.8	198	18.9
*Iron therapy type % of treated patients*										
*IV iron*	46	47.4	55	79.7	18	66.7	1	1	120	60.6
*Oral iron*	51	52.6	14	20.3	9	33.3	4	3.8	78	39.4
*Erythropoiesis-stimulating agents (ESA)*	51	16.4	21	5.7	30	11.5	4	3.8	106	10.1

ESAs were used during chemotherapy in 104 (10.8%) patients with Hb below 10 g/dL, including 3 patients with Hb between 7 and 8 g/dL and 3 patients with Hb below 7 g/dL. In the subgroup without ID (*n* = 263), 43 (16.3%) patients received ESA therapy. In the subgroup without functional ID (TSAT > 20%, *n* = 52), 19 (36.5%) patients received ESA therapy; these were only patients with chemotherapy and Hb between 8 and 10 g/dL.

Vitamin B12 was administered in 60 patients (5.7%).

### Guideline adherence scores: diagnosis (GLAD-D), therapy (GLAD-T)

The results of guideline adherence and GLAD scores are shown in Table 3.

**Table 3:** GLAD scores for anemia diagnostics (GLAD-D) and therapy (GLAD T)

GLAD-D (diagnostic)	0		1		2	
	*N*	%	*N*	%	*N*	%
Breast cancer	189	60.8	36	11.6	86	27.7
Gastrointestinal cancer	174	46.9	78	21.0	119	32.1
Lung cancer	153	58.8	32	12.3	75	28.8
Malignant lymphoma	52	50.0	22	21.2	30	28.8
Total	568	54.3	168	16.1	310	29.6
GLAD-T (therapy)	0		1		2	
	*N*	%	*N*	%	*N*	%
Breast cancer	118	37.9	84	27.0	109	35.0
Gastrointestinal cancer	160	43.1	126	34.0	85	22.9
Lung cancer	120	46.2	72	27.7	68	26.2
Malignant lymphoma	58	55.8	38	36.5	8	7.7
Total	118	37.9	84	27.0	270	25.8

In another finding, there was a positive correlation between guideline-adherent differential diagnosis and guideline-adherent anemia treatment: 45.2% of guideline-adherent diagnosed patients (GLAD-D 2) received guideline-adherent treatment, whereas only 17.4% of poorly diagnosed patients (GLAD-D 0 and 1) received guideline-adherent treatment (OR 4.41; 95% CI 3.17–6.17; *p* < 0.001).

In 198 of all 1046 patients, who received iron replacement, 54.5% had a GLAD-D 2, while 23.8% (*p* < 0.001) of the remaining 848 patients without iron replacement had a GLAD-D 2. In the 120 patients receiving IV iron replacement, the GLAD-D 2 was 60.8% compared to 44.9% for oral therapy (*p* = 0.016).

**Fig. 2:**
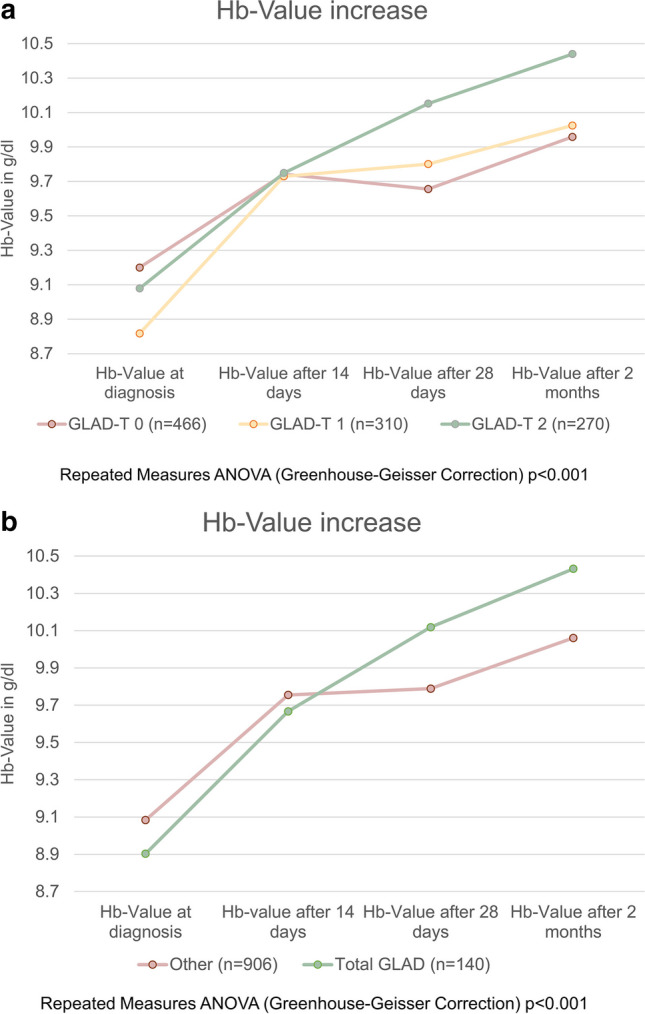
**a** Anemia therapy, GLAD score T and increase of Hb value after anemia diagnosis. **b** Anemia therapy, total GLAD (GLAD D + GLAD T) vs. other and increase of Hb value after anemia diagnosis

Guideline-directed therapy of anemia was associated with a significantly faster and, more importantly, more sustained increase in Hb levels at both the 28-day and 2-month time points (GLAD-T 0: 9.65 g/dL and 9.96 g/dL, respectively, vs. GLAD-T 2 10.15 g/dL and 10.44 g/dL, respectively); see also Fig. 2a. Hb levels increased by 1.07 g/dL and 1.3 g/dL for GLAD-T 2 absolute at 4 weeks and 2 months compared to the combined GLAD-T 0/1 by 0.46 g/dL and 0.76 g/dL, respectively.

The summary of GLAD scores of diagnosis and therapy was defined as total GLAD score. The increase in Hb at 28 days and 2 months was significantly higher in patients with a total GLAD 2 compared to total GLAD 0/1: 10.12 g/dL and 10.43 g/dL compared to 9.79 g/dL and 10.06 g/dL, respectively; see Fig. 2b. At 28 days and 2 months, the absolute differences for total GLAD 2 were 1.22 g/dL and 1.53 g/dL, respectively, compared with 0.71 g/dL and 0.98 d/dL for total GLAD 0/1.

Two multivariable models were developed to examine the extent to which patient characteristics and health care parameters correlated with guideline-directed anemia therapy. Since there was little difference in Hb increase between patients with GLAD-T 0 and GLAD-T 1, they were combined here so that guideline-directed therapy (GLAD-T 2) was compared with non-guideline-directed therapy (GLAD-T 0/1). The following patient characteristics were significant when comparing odds ratios.

**Fig. 3:**
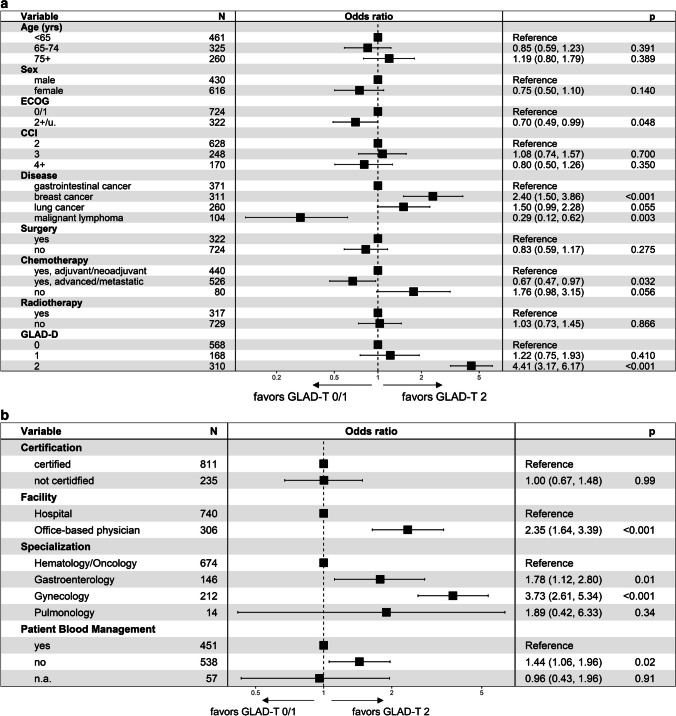
**a** Multivariable model, patient and treatment characteristics and GLAD score T. **b** Multivariable model, center characteristics and GLAD score T

Significantly worse GLAD-T was seen with an ECOG score of 2 and higher, malignant lymphoma, chemotherapy for advanced or metastatic disease. Significantly better GLAD-T was seen in breast cancer, patients without chemotherapy and a GLAD-D of 2; see Fig. 3a.

Patients who did not undergo surgery or underwent minimally invasive procedures were slightly less likely to receive guideline-directed anemia therapy (22.5%) than patients who underwent surgery (33.2%). This difference was not significant in the multivariable model: OR 0.83 (95% CI 0.59–1.17, *p* = 0.275); see below. Patients with no medical tumor therapy were slightly more likely to receive guideline-directed anemia therapy (37.5%) than patients in the adjuvant or neoadjuvant setting (31.1%): OR 1.76 (95% CI 0.98–3.15), but the difference was not significant at the given level of significance (*p* = 0.056). However, patients with advanced or metastatic disease were significantly less likely to receive guideline-directed anemia therapy (19.6%): OR 0.67 (95% CI 0.47–0.97, *p* = 0.032). In contrast, therapy did not differ between patients with and without radiotherapy: OR 0.97 (95% CI 0.69–1.36, *p* = 0.849).

Regarding center parameters, a higher proportion of patients in practices/health centers (34.6%) were treated with guideline-directed anemia therapy than in clinics (22.2%): OR 2.42 (95% CI 1.70–3.48, *p* < 0.001).

The multivariable analysis is shown in Fig. 3b, with a significantly better GLAD therapy score when no patient blood management program existed in the institution.

The proportion was also higher in organ-specific institutions (gynecology, gastroenterology, pulmonology) (36.0%) than in hematology-oncology institutions (20.2%): gynecology OR 3.81 (95% CI 2.68–5.45, *p* < 0.001), gastroenterology OR 1.84 (95% CI 1.84–2.88, *p* = 0.009), and pneumology OR 1.91 (95% CI 0.42–6.39, *p* = 0.333). However, it should be noted that many organ-specific centers are multidisciplinary. There was no statistically significant difference between certified centers (25.1%) and non-certified centers (28.0%): OR 0.96 (95% CI 0.65–1.42, *p* = 0.854).

## Discussion

Diagnostic workup is a prerequisite for adequate anemia therapy. Appropriate workup parameters for iron, vitamin B12, and folate deficiency are provided by ESMO and other guidelines [1, 16, 31].

In the present study, the median Hb level of all patients was 9.3 g/dL, with minor differences between the diseases studied. Since ferritin may be elevated in cancer patients and is not a reliable parameter of iron status, serum transferrin saturation should be determined. This parameter can also diagnose anemia of chronic disease or inflammation, which is common in cancer patients [20]. In routine clinical practice, transferrin saturation is determined in only 14% of patients throughout Europe [35]. Unfortunately, this has hardly improved since 2014: in our study transferrin saturation was determined in only 19.5% of patients overall and in 16.9% and 15.4% of patients with lung cancer and malignant lymphoma, respectively. Patients with gastrointestinal cancers had the highest rate of testing at 22.1%. Of the 125 patients who were initially screened, 62.4% had a decreased TSAT. The prevalence of ID, diagnosed by decreased transferrin saturation below 20%, was 42.6% in tumor patients and 33% of all patients were anemic [36].

Other parameters of ID such as percentage of hypochromic erythrocytes or soluble transferrin receptor did not play a role with an application rate of 3.2% and 1.2%, respectively.

For the diagnosis of ID as a common cause of anemia, the MCV value can be used, but the correlation with ID is not good, so other parameters are preferred [4, 21]. In the tumor patients studied here, anemia is more likely to occur in chronic diseases, in which MCV can be both normal and decreased [12, 50]. In 19.6% of our patients, the MCV value was below 80 fl, indicating prolonged ID.

In our study, the simple and convincingly published determination of reticulocyte hemoglobin as a parameter for the iron status of erythropoiesis was used in only 6.2% of patients [4, 13, 42, 45, 49].

Although TSAT and reticulocyte hemoglobin were only determined in a small proportion of patients in routine clinical practice, we found a significant correlation between TSAT and reticulocyte hemoglobin. Thus, we can confirm the detection of iron-deficient erythropoiesis by means of decreased reticulocyte hemoglobin [4, 42, 45, 49].

Certainly, it is not enough to look at individual parameters to differentiate ID [4, 9, 18].

Even without the classical parameter of an increased erythrocyte MCV, a deficiency of vitamin B12 or folic acid may be present [41] so that these should be determined directly. Vitamin B12 and folic acid diagnostics were performed in only 8.1% and 7.4% of the patients, respectively.

If only patients with hemoglobin levels below 8.0 g/dL received blood transfusions, the number of RBC transfusions could be significantly reduced. This group could be further reduced if, according to guidelines, only patients with risk factors or anemic hypoxia received transfusions.

Even in hematologic patients, one transfusion compared to two transfusions is not detrimental to the patient [7, 15]. A restrictive transfusion strategy (Hb concentration 7–9 g/dL) compared to a liberal transfusion strategy (Hb concentration 8 to 12 g/dL) showed little or no difference in mortality at day 30 to 100, length of hospital stay, or frequency of bleeding complications [19]. The guidelines recommend to transfuse only one unit if possible, but in the study, only 28.3% of the RBC administrations transfused one unit.

However, it is unclear whether the requirement of only one transfusion is feasible in the practice of outpatient anemia therapy, as one must weigh the disadvantages of possibly more frequent transfusions and the time involved [17].

A large percentage of RBCs were transfused without indication, i.e., at Hb values between 7 and 8 g/dL without risk factors or between 8 and 10 g/dL without anemic hypoxia, corresponding to 287 (36.6%) of 785 patients without transfusion indication. Also, in the previous Europe-wide analysis by Ludwig, a very large number of patients (29.6%) received RBC transfusions at Hb values between 9 and 9.9 g/dL [37].

Not only in surgical specialties, but also in oncology, a patient blood management (PBM) program, i.e., standardized anemia diagnosis and therapy, decreases the transfusion rate in both the inpatient and outpatient sectors [24]. Our study showed that the GLAD-T score was better in the absence of a PBM program. Whether this result is due to a lack of knowledge about PBM or only related to a PBM of other hospital departments would require further investigations.

A PBM program also means optimizing anemia diagnosis and therapy, including transfusion practices. This can optimize preoperative Hb levels and reduce transfusion rates, ICU admissions, length of hospital stay, and overall complications [10, 25, 40]. The negative effect of transfusion on cancer recurrence could also be avoided [29]. Recent analyses also show a worse effect of immunotherapy in transfused patients [39].

In case of absolute or functional ID, patients with existing tumor should be substituted with IV iron, as most of the time the immune system is stimulated with corresponding interleukin-6 and hepcidin production, blocking oral iron uptake and iron release from stores [1, 11, 20]. In our study, we used elevated CRP as a parameter for interleukin-6 production. In 24.4% of the patients, this correlation was ignored, and they were treated with oral iron. IV iron therapy is safe [8], whereas red blood cell transfusions are associated with a tenfold higher risk of severe morbidity than IV iron (1 in 21,413 for RBC versus 1 in 200,000 for actual IV iron products) [6].

In the EU, ESA therapy is approved and recommended by guidelines for patients with Hb ≤ 10 g/dL receiving chemotherapy (https://www.ema.europa.eu/) [1, 16, 44].

When ESAs are used with chemotherapy, there is no evidence of a negative impact on survival or progression-free survival [2].

With only 10.8% of patients treated, ESA therapy did not play a major role. Considering only the 263 patients with chemotherapy and Hb levels between 8 and 10 g/dL and a clear indication for ESA therapy, i.e., without ID, 43 (16.3%) received ESA therapy. Only in the small subgroup with measured TSAT and values above 20%, 19 (36.5%) of 52 patients received ESAs.

We showed that with a guideline-concordant diagnosis and therapy, i.e., our total GLAD score of 2, the Hb level increased significantly more at 4 weeks and 2 months compared to a score of 0/1. Even if only the GLAD-T 2 of the therapy is considered, the results are better than with a score of 0/1.

This confirms that the goal of anemia therapy can be achieved significantly better with consistent guideline adherence.

Our multivariable analysis revealed significant factors for a GLAD-T 2. Not surprisingly, an ECOG score of 2 and higher, as patients with advanced disease and likely shorter life expectancy have little benefit from strict guideline adherence. Interestingly, the guidelines were followed much less frequently in patients with malignant lymphoma or chemotherapy. There were also significant differences between hospital and office-based physicians and between specialties in favor of gastroenterology and gynecology.

Limitations of the study are the retrospective methods, no subsequent research could be conducted at the participating institutions, and the possible non-participation of particularly motivated institutions or even guideline opponents. It is possible that not all arguments and reasons for decisions regarding therapy and particularly transfusions are documented in the medical records. In addition, patient preferences in terms of shared decision-making were not reliably captured.

The low rate of guideline implementation is likely due to several factors, such as exaggerated concern about the toxicity of intravenous iron or ESAs, possibly insufficient knowledge of the pathophysiology of anemia in cancer, ignoring the toxicity and long-term consequences of RBC transfusions, and insistence on previous but outdated therapeutic principles. To disseminate and implement guidelines in oncology, reminders and feedback on guideline adherence seem to be effective [46]. The use of rigorous guidelines and clinical pathways has improved implementation of transfusion guidelines [22, 23]. However, it is unclear whether a clinical decision system can also reduce the rate of RBC transfusion in hematology and oncology [5].

In conclusion, the implementation of the European and German anemia guidelines for diagnosis and therapy in Germany is not sufficient, and effective measures must be taken to transfer them into clinics and practices.

## Data Availability

The funding source had no access to the data and was not involved in data analysis or writing of the manuscript. Authors confirm that they have full control over all primary data and agree to allow the journal to review their data if requested.

## Declarations

### Standard of reporting

The guidelines for Observational Studies in Epidemiology (STROBE) were taken into account as far as applicable [47].
